# Phylogenomics of asexual *Epichloë* fungal endophytes forming associations with perennial ryegrass

**DOI:** 10.1186/s12862-015-0349-6

**Published:** 2015-04-24

**Authors:** Inoka K Hettiarachchige, Piyumi N Ekanayake, Ross C Mann, Kathryn M Guthridge, Timothy I Sawbridge, German C Spangenberg, John W Forster

**Affiliations:** Department of Economic Development, Jobs, Transport and Resources, Biosciences Research Division, AgriBio, Centre for AgriBioscience, Bundoora, Melbourne, Victoria 3083 Australia; School of Applied Systems Biology, La Trobe University, Bundoora, Melbourne, Victoria 3086 Australia; Molecular Plant Breeding Cooperative Research Centre, Melbourne, Victoria 3083 Australia; Dairy Futures Cooperative Research Centre, Melbourne, Victoria 3083 Australia

**Keywords:** Pasture grass, Whole genome sequencing, Taxonomy, Nuclear gene, Mating type, Progenitor

## Abstract

**Background:**

Perennial ryegrass (*Lolium perenne* L.) is one of the most important species for temperate pastoral agriculture, forming associations with genetically diverse groups of mutualistic fungal endophytes. However, only two taxonomic groups (*E. festucae* var. *lolii* and *Lp*TG-2) have so far been described. In addition to these two well-characterised taxa, a third distinct group of previously unclassified perennial ryegrass-associated endophytes was identified as belonging to a putative novel taxon (or taxa) (PNT) in a previous analysis based on simple sequence repeat (SSR) marker diversity. As well as genotypic differences, distinctive alkaloid production profiles were observed for members of the PNT group.

**Results:**

A detailed phylogenetic analysis of perennial ryegrass-associated endophytes using components of whole genome sequence data was performed using complete sequences of 7 nuclear protein-encoding genes. Three independently selected genes (encoding a DEAD/DEAH box helicase [Sbp4], a glycosyl hydrolase [family 92 protein] and a MEAB protein), none of which have been previously used for taxonomic studies of endophytes, were selected together with the frequently used ‘house-keeping’ genes *tefA* and *tubB* (encoding translation elongation factor 1-α and β-tubulin, respectively). In addition, an endophyte-specific gene (*perA* for peramine biosynthesis) and the fungal-specific *MT* genes for mating-type control were included. The results supported previous phylogenomic inferences for the known species, but revealed distinctive patterns of diversity for the previously unclassified endophyte strains, which were further proposed to belong to not one but two distinct novel taxa. Potential progenitor genomes for the asexual endophytes among contemporary teleomorphic (sexual *Epichloë*) species were also identified from the phylogenetic analysis.

**Conclusions:**

Unique taxonomic status for the PNT was confirmed through comparison of multiple nuclear gene sequences, and also supported by evidence from chemotypic diversity. Analysis of *MT* gene idiomorphs further supported a predicted independent origin of two distinct perennial ryegrass-associated novel taxa, designated *Lp*TG-3 and *Lp*TG-4, from different members of a similar founder population related to contemporary *E. festucae.* The analysis also provided higher resolution to the known progenitor contributions of previously characterised perennial ryegrass-associated endophyte taxa.

**Electronic supplementary material:**

The online version of this article (doi:10.1186/s12862-015-0349-6) contains supplementary material, which is available to authorized users.

## Background

The genus *Lolium,* which belongs to the sub-family Pooideae (cool-season grasses) of the grass and cereal family Poaceae, includes several important forage and turf species [1,2]. Perennial ryegrass (*Lolium perenne* L.) is extensively cultivated for pasture production on a global basis [3,4]. Like other cool-season grasses, perennial ryegrass is often infected with clavicipitaceous fungal endophytes that include both sexual and asexual taxa [5-7]. The asexual (anamorphic) taxa were previously assigned to a separate genus (*Neotyphodium*), but in accord with general recommendations for fungal taxonomy, have been recently been combined with the sexual (teleomorphic) taxa within a single genus, designated *Epichloë* [8]. Asexual *Epichloë* endophytes colonise the intercellular spaces of leaf sheaths, culms, and rhizomes, and infrequently the surface of leaf blades, without inducing obvious pathological symptoms [9]. These asexual endophytes do not produce stromata, and rely solely on the host plant for transmission [10,11]. The vegetative phase of growth for sexual is similar to that of asexual *Epichloë* species [12], but in the sexual stage, stromata are formed around the developing inflorescences and prevent emergence of the floral meristem [7,12].

Asexual *Epichloë* species form mutualistic associations with their hosts [13,14]. Benefits for the host related to abiotic stress tolerance are obtained through enhanced growth, increased seedling vigour and persistence, particularly under water stress and nutrient deficiency [15,16]. The endophyte also confers biotic stress tolerance to the host grass, through production of several classes of biologically active alkaloids. Peramine and loline produced by the endophyte are active against insect pests, while lolitrem B and ergovaline are toxic to mammalian herbivores [17-19]. Conversely, as part of the symbiosis, the plant provides certain benefits to the endophyte such as shelter, nutrition, reproduction and distribution [14,20,21].

Perennial ryegrass has been found to be host to two distinct fungal endophyte taxa: *Epichloë festucae* var. *lolii* (Latch, Christensen and Samuels) Bacon and Schardl syn. *N. lolii* (Latch, Christensen and Samuels) Glenn, Bacon and Hanlin [22] and *Lolium perenne* taxonomic group 2 (*Lp*TG-2) [23]. Distinct taxonomic groups of asexual *Epichloë* endophytes are proposed to have evolved either by direct evolution from a single teleomorphic species, probably due to loss of the sexual state, or through interspecific hybridisation events between either sexual species or distinct sexual and asexual lineages, the latter generating heteroploid genetic constitutions [24-27]. The haploid taxon *E. festucae* var. *lolii* has been identified as a direct derivative of *Epichloë festucae* Leuchtm., Schardl & M. R. Siegel while the heteroploid *Lp*TG-2 arose as an interspecific hybrid between *E. festucae* var. *lolii* and *E. typhina* (Pers.) Tul. & C. Tul. [24,26].

Previous phylogenetic characterisation studies of both sexual and asexual *Epichloë* species have largely been based on the use of partial sequences of genes encoding highly conserved proteins such as *tefA*, *tubB* and *act1* (actin), including both relatively invariant coding sequences and more diverse intronic regions [25,28,29]. In addition, sequence diversity of the genes responsible for alkaloid biosynthesis has also been employed for phylogenetic analysis. Specifically, the *perA* gene, which is responsible for peramine production, has been identified as potentially exhibiting a higher rate of molecular evolution than ‘house-keeping’ genes, permitting resolution of close taxonomic relationships [30]. Gene sequences of *perA*, along with several of the more conserved genes, have previously been used to study relationships between endophytes of perennial ryegrass and members of the closely related grass genus, *Festuca* (fescues) [30,31], including elucidation of the ancestral lineages that have contributed to contemporary hybrid endophytes [32]. In addition, use of different approaches such as morphological and physiological variation of cultured endophytes, isoenzyme variation and gene-based sequence analysis have been used to define and promote new taxonomic groups [33]. Due to advances in genomic technologies, complete sequences from various genes have increased the reliability of phylogenetic inference [30]. However, differences in performance for phylogenetic studies have been reported between individual genes [1]. This limitation may be addressed through the use of multiple gene sequences, as a proxy for variation across the entirety of the endophyte genome.

Molecular genetic markers based on sequence polymorphism may also be used to address issues of endophyte diversity, taxonomy and phylogeny [1]. A large set of SSR marker loci has been developed for the identification and assessment of genetic diversity among asexual *Epichloë* endophytes [34]. In previous studies of perennial ryegrass-derived endophyte diversity based on variation of 18 such SSR markers, three distinct groups of perennial ryegrass endophytes were observed [35]. In addition to the well-characterised taxa, *E. festucae* var. *lolii* and *Lp*TG-2, a third distinct group of previously unclassified endophytes was identified as belonging to one or more PNT. However, SSR variation, although indicative, is of only limited value for definition of taxonomic groupings, due to the generation of data suitable for phenetic, rather than phylogenetic, analysis. Nonetheless, putative novel taxa of fescue-derived heteroploid endophytes that were predicted on the basis of SSR-based phenetic dendrograms [36] were demonstrated to be genuinely distinct on the basis of subsequent phylogenomic studies [30].

The present study describes the evolutionary relationships between 19 representative perennial ryegrass-associated endophyte strains belonging to the known taxa *E. festucae* var. *lolii* and *Lp*TG-2, and the uncharacterised PNT, as well as several putative ancestral sexual *Epichloë* species. The phylogenetic analysis was based on 7 full-length gene sequences derived from whole genome sequence datasets, including *tefA*, *tubB* and *perA* which were previously used for a corresponding study of fescue-derived endophytes [30]. In addition, complete sequences of three further independently selected nuclear genes (for which full-length sequence coverage is available for all strains analysed) were included: DEAD/DEAH box helicase (Sbp4), glycosyl hydrolase (family 92 protein) and MEAB protein. DEAD/DEAH box helicases are a family of proteins which are involved in various processes of RNA metabolism [37]. Glycoside hydrolases are enzymes present in a wide range of organisms which hydrolyse the glycosidic bond between a carbohydrate and another compound, such as a second carbohydrate, a protein, or a lipid [38]. MEAB is a protein that mediates nitrogen catabolite repression in fungi [39]. Finally, the mating type (*MT*) genes, which play an important role in evolution of fungal species through control of sexual compatibility, were also included.

## Methods

### Selection of endophyte strains

A total of 19 endophyte strains that form associations with perennial ryegrass were used for the phylogenetic analysis (Table 1). The genetic properties [40] and phenotypic [39] properties of NEA2, NEA3 and NEA4 were formerly described. All other strains were as previously defined [35], apart from E1, which was originally isolated from an accession of chewings fescue (*F. rubra* ssp. *commutata* Gaudin). However, E1 was shown to be capable of stable establishment after introduction into multiple genotypes of perennial ryegrass through artificial inoculation into regenerating meristem-derived callus [33] (data not shown). E1 displays a closer similarity to previously described PNT genotypes such as NEA12 than to representatives of *E. festucae* var. *lolii* and *Lp*TG-2, and so was included in this study, but is nonetheless distinct in genetic terms [35].

**Table 1:** **Perennial ryegrass-associated endophyte isolates used in this study**

**Species/Taxon**	**Strain/isolate ID**	**Origin**	**Source**
***E. festucae*** **var.** ***lolii***	SE	New Zealand	DEDJTR
	15335	Italy	DEDJTR
	15441	Italy	DEDJTR
	15714	Turkey	DEDJTR
	NEA3	Romania	NZA
	F02	Spain	NZA
	AR1	Italy	AgResearch New Zealand
	C09	Spain	NZA
	E09	Former Soviet Union	NZA
	NA6	Morocco	DEDJTR
	NEA10	Spain	NZA
	15931	Former Soviet Union	DEDJTR
	NEA2	Spain	NZA
**PNT**	15310	France	DEDJTR
	15311	France	DEDJTR
	E1	(F. rubra ssp. *commutata*)	RBG
	NEA12	France	DEDJTR
***Lp*** **TG** ***-*** **2**	NEA4	France	NZA
	NEA11	France	RBG

DEDJTR: Department of Economic Development, Jobs, Transport and Resources, Victoria, Australia; NZA: New Zealand Agriseeds Limited, Christchurch, New Zealand; AgResearch New Zealand, Hamilton, New Zealand; RBG: Royal Barenbrug Group, Nijmegen, Netherlands.

### Generation and assembly of genome sequence data

Genomic paired-end reads generated by sequencing libraries prepared using the TruSeq™ low-throughput (LT) DNA sample preparation kit (Illumina Inc, San Diego, California, USA) were used to generate assemblies of ryegrass-associated endophyte genomes. Low-quality sequence reads were filtered out using a custom python script, which calculates quality statistics and stores trimmed reads in several fastq files. Genome assemblies of endophytes C09, E1, NEA4 and NEA11 were obtained through VelvetOptimiser (version 2.2.0) using default parameters except for hash length (K-mer sizes) which was in the range of 31 to 71. Previously generated genome assemblies from other endophyte strains [40,41] were used in this analysis.

### Identification of gene sequences

Presence and copy number of *tefA*, *tubB, perA*, DEAD/DEAH helicase, glycosyl hydrolase, MEAB protein and *MT* genes in each endophyte genome were initially identified through nucleotide BLAST (Basic Local Alignment Search Tool) (version 2.2.25) analysis [42] using contigs from the optimised velvet assembly of total reads as the database which was queried with the following reference sequences (Genbank accession numbers: *tubB*: AY722412 from *E. festucae* Fl1; *tefA*: FJ660614 from *E. festucae* E2368; *perA*: AB205145 from *E. festucae* Fl1; glycosyl hydrolase, family 92 protein: XM_006669157.1 from *Cordyceps milit*aris CM01; DEAD/DEAH box helicase (Sbp4): XM_007820576.1 from *Metarhizium anisopliae* ARSEF 23; MEAB protein: XM_007825059.1 from *M. anisopliae* ARSEF 23; *MT* genes: FJ717711.1 (*mtAA*), HQ680588.1 (*mtAB*), HQ680587.1 (*mtAC*) from *E. festucae* E2368 and HQ680590.1 (*mtBA*) from *E. festucae* Fl1. Following identification of contigs and gene coordinates for selected genes within the genomes of the haploid strains (*E. festucae* var. *lolii* and PNT) using corresponding reference genes, the contig and subsequently, the relevant gene sequence was extracted using fastacmd (version 2.2.24) (www.ncbi.nlm.nih.gov) and EMBOSS extractseq (version 6.3.1) (gwilliam@hgmp.mrc.ac.uk), respectively. A similar approach was used to extract corresponding gene sequences from the genomes of *E. typhina* 9340, 9636 and *E. baconii* 9707 based on the relevant databases. Data for representative *Epichloë* species isolates was extracted from genome sequence data available at http://www.endophyte.uky.edu/ [43].

A different approach was used to extract genes from heteroploid strains (*Lp*TG-2), due to the presence of two copies for each gene. Corresponding gene sequences of the predicted progenitors, *E. festucae* var. *lolii* (SE) and *E. typhina* (9636) were used as references for mapping of all sequencing reads from *Lp*TG-2 strains using Bowtie (version 1.0.0) (Langmead). Mapped reads were assembled using velvet (version 1.1.06) [44] using the corresponding gene as the reference to obtain two copies for each *Lp*TG-2 strain.

### Phylogenetic analysis

Sequences of all selected endophyte strains were aligned using MUSCLE, built-in version of MEGA (version 5.2.2) [45]. Alignments were checked manually for ambiguities and adjusted if necessary. To construct the tree topology, maximum likelihood (ML) and neighbour-joining (NJ) methods were used as implemented in MEGA 5.2.2 with default parameters. Positions containing gaps or missing data were eliminated in constructing phylogenetic trees. Robustness of inferred phylogenies was estimated by bootstrap replication (1,000 replicates), and branches receiving less than 60% of replicates were collapsed.

For the sake of clarity, gene copies of *Lp*TG-2 strains were appended with the suffixes GC1 and GC2, reflecting phylogenetic affinity with *E. festucae* var. *lolii* and *E. typhina*, respectively. Megablast searches [40] using *tubB*, *tefA* and *perA* sequences were performed in GenBank nucleotide sequence database of the National Centre for Biotechnology Information (NCBI) to identify the closest matching sequences of *Epichloë* species, which were added to the sequence alignment. Accession numbers for GenBank-derived sequences are provided in each phylogram next to the isolate name.

Phylogenetic analysis was also performed with the concatenated sequences. The sequences of all, except *MT*, genes were concatenated in the order of *tefA*, *tubB*, *perA*, DEAD/DEAH helicase, glycosyl hydrolase and MEAB protein into a multi-gene alignment which was then aligned using MUSCLE. Phylogenetic topology was constructed using the same methods as described above.

## Results

### Assembly of genome sequence data

Statistics for genome assemblies of perennial ryegrass-associated endophyte strains are provided in Additional file 1.

### Identification of nuclear gene copies

BLASTN analysis, as described, was used to identify the presence of the 7 selected gene sequences in the genomes of all candidate strains and reference sexual *Epichloë* species. Single copies of each gene were identified for all isolates of *E. festucae* var. *lolii* and PNT, reflecting haploid genome structures. In contrast, two copies of each gene were observed for both representatives of taxon *Lp*TG-2. Complete copies of the *perA* gene were identified in *E. festucae* var. *lolii* and *Lp*TG-2 strains, but all PNT representatives showed the presence of a 1223 bp deletion at the 3’-end of the gene. Furthermore, apart from strain E1, all PNT strains contained a premature stop codon at coordinate 2923 bp within the *perA* gene sequence.

### Phylogenetic characterisation based on individual gene sequences

Phylograms were constructed on an individual gene basis using the ML and NJ methods. In all instances, except for the *MT* genes, the phylogram structure revealed three major distinct sequence clades. The single gene copies from *E. festucae* var. *lolii* and GC1 from *Lp*TG-2 were located in the first clade, while *Lp*TG-2 GC2 was located in the second clade. For all phylograms, the third clade contained single gene copies from PNT representatives, except for that generated from *tefA* sequences, in which strain E1 was grouped with the *E. festucae* var. *lolii* strains in clade 1. The separation of the three clades was supported by >80% bootstrap values in all instances (results not shown). For *Lp*TG2-derived sequences, lower variability was observed between GC1 representatives from the alternate strains than for those from GC2.

**Figure 1:**
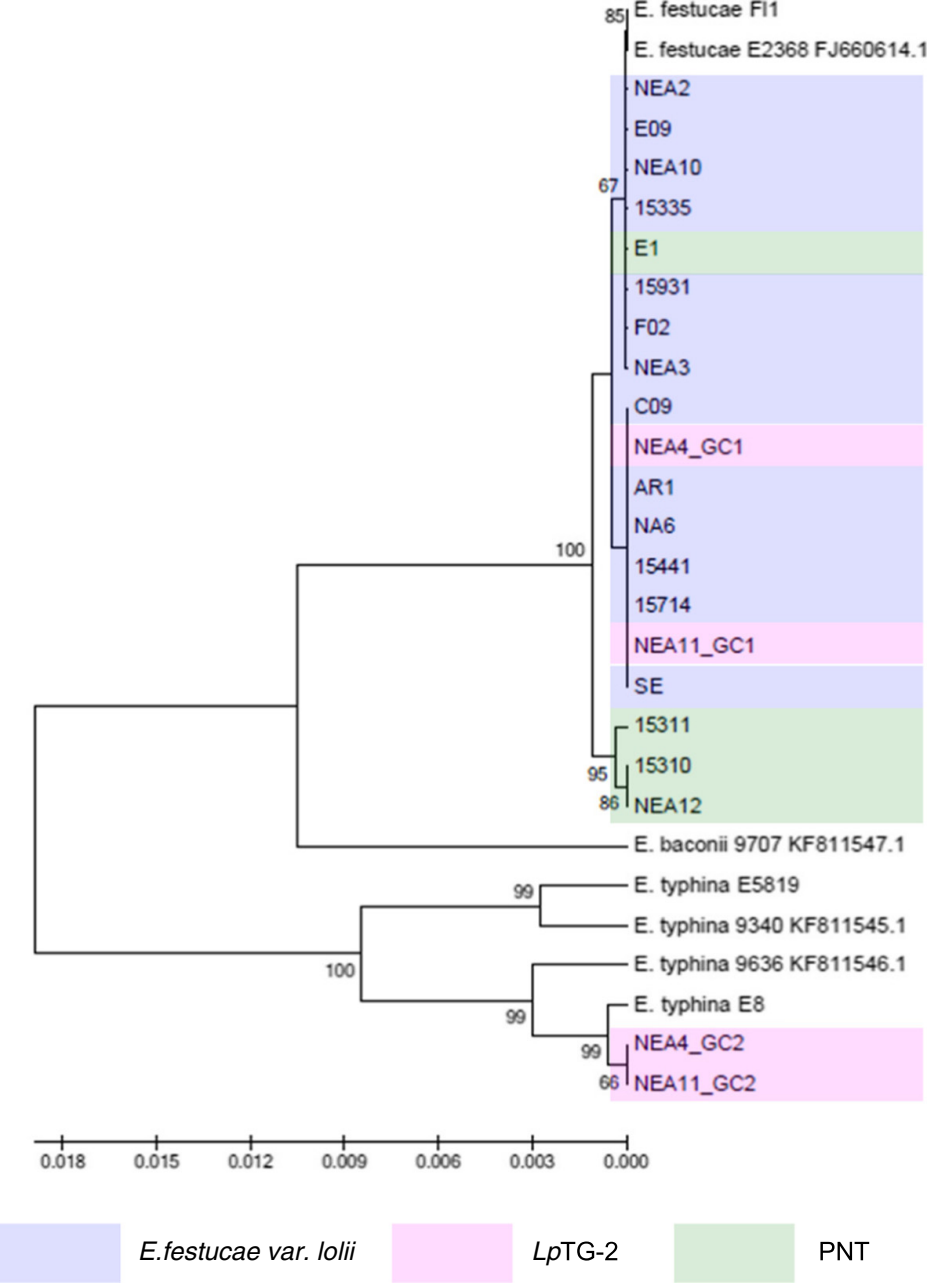
Phylogram resulting from ML analysis of *tefA* gene sequences of selected perennial ryegrass-associated endophytes and reference isolates, with bootstrap values (>60% from 1,000 replicates) shown on nodes. Endophyte taxa are colour-coded as indicated in the legend. The GenBank accession numbers of the *tefA* genes derived from ryegrass-associated endophytes are provided in Additional file 4, and GenBank accession numbers of the reference sequences are provided following taxon and strain identities.

**Figure 2:**
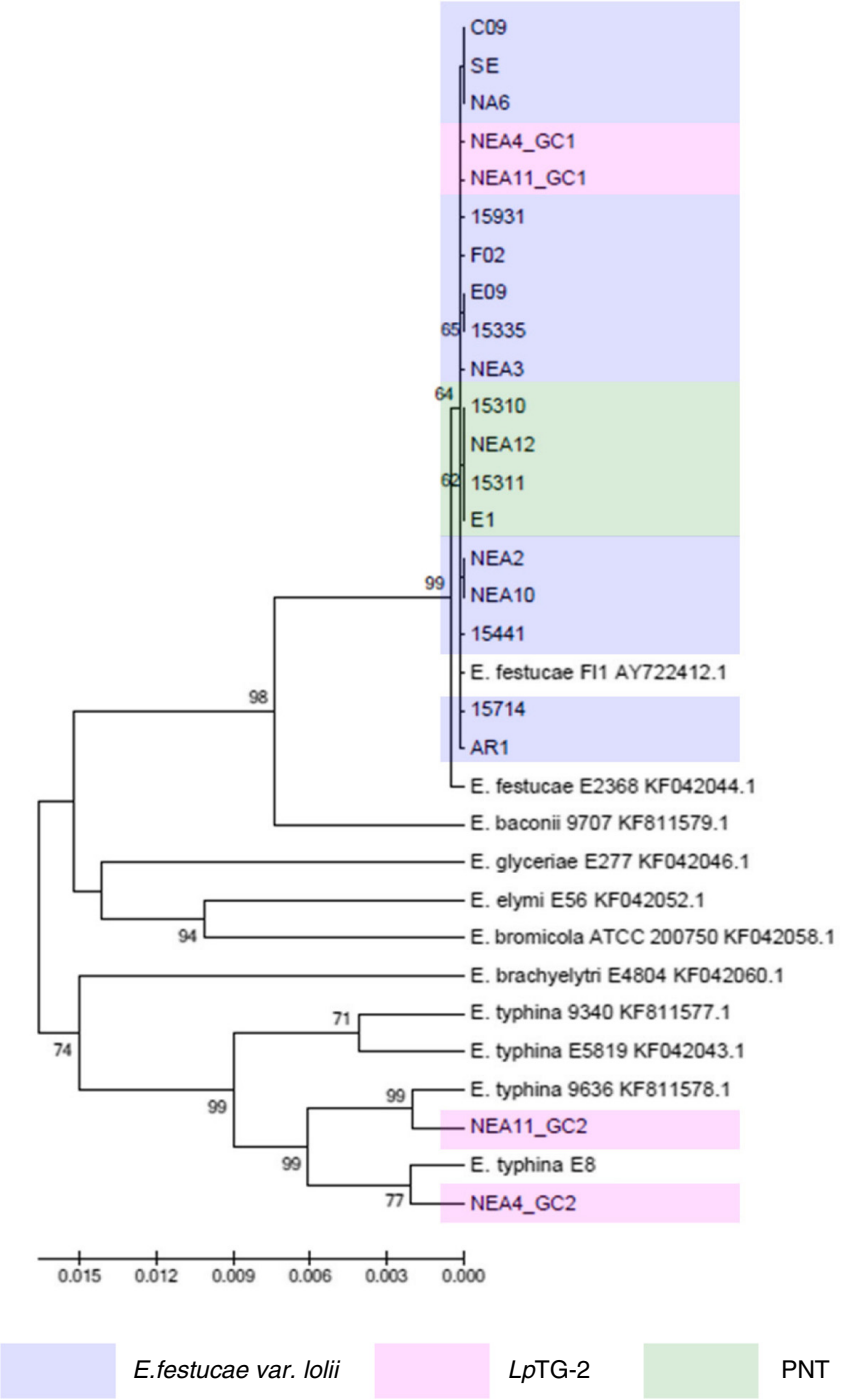
Phylogram resulting from ML analysis of *tubB* gene sequences of selected perennial ryegrass-associated endophytes and reference isolates. Diagram properties are as described for Figure 1.

**Figure 3:**
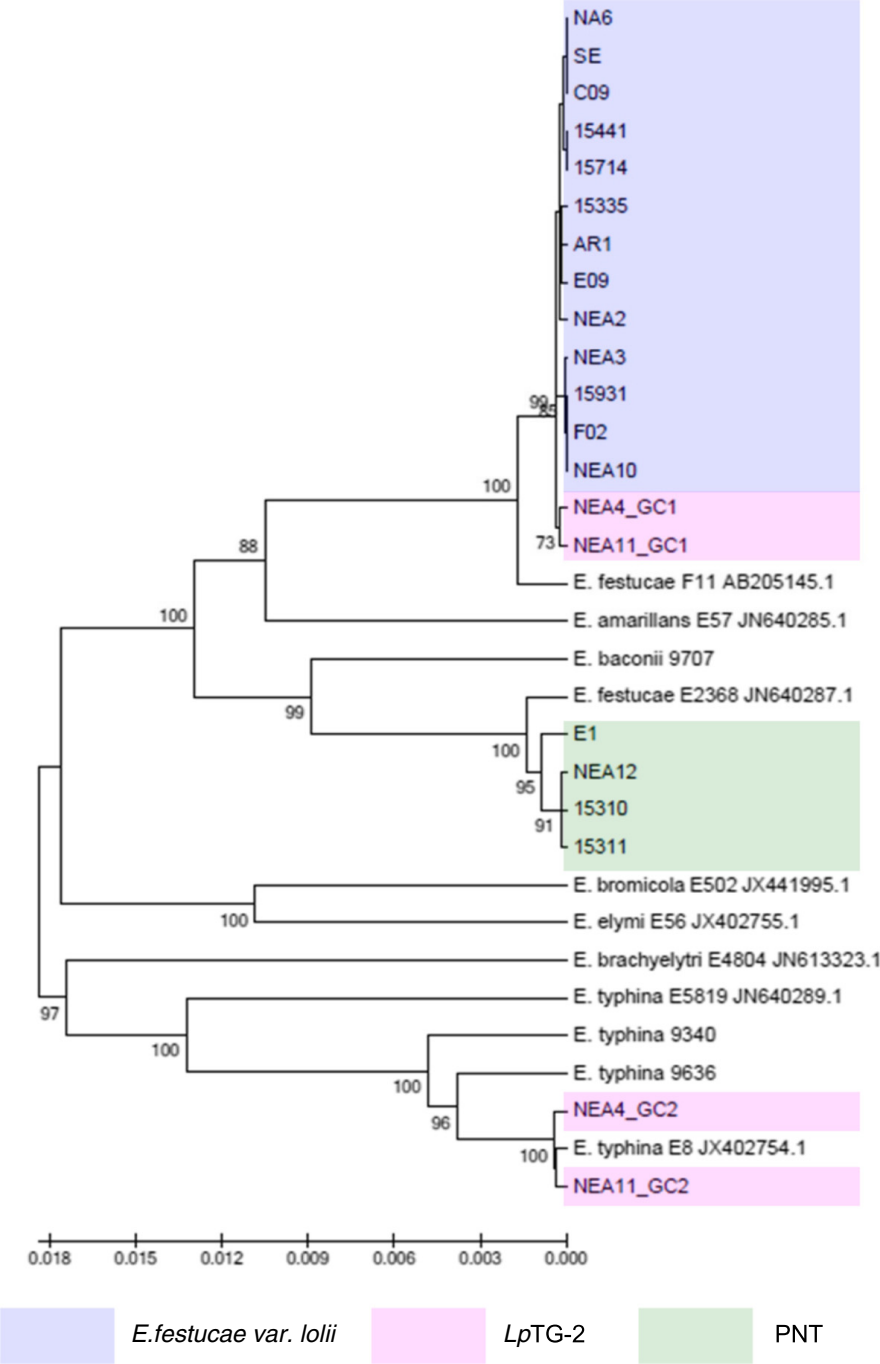
Phylogram resulting from ML analysis of *perA* gene sequences of selected perennial ryegrass-associated endophytes and reference isolates. Diagram properties are as described for Figure 1.

**Figure 4:**
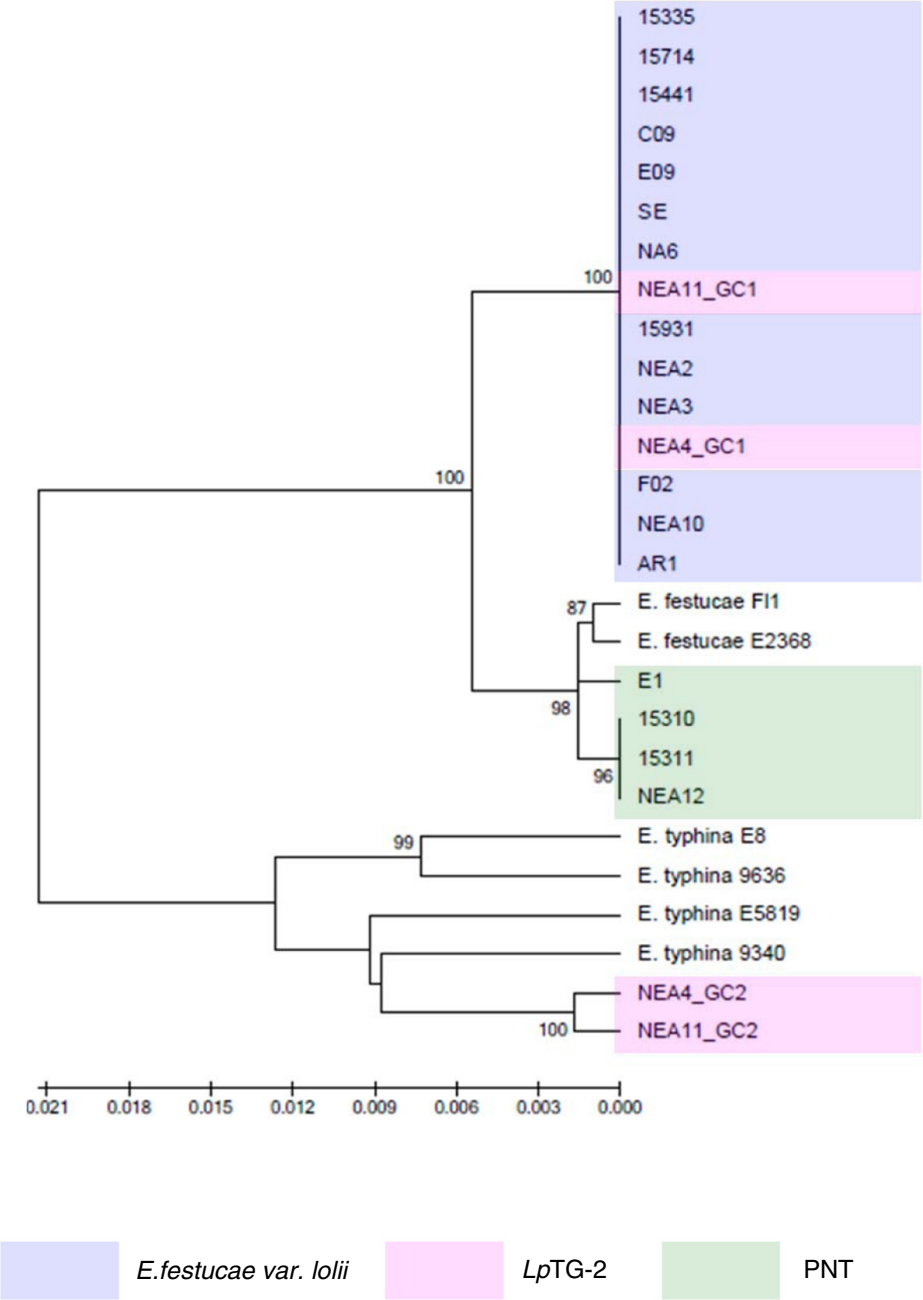
Phylogram resulting from ML analysis of glycosyl hydrolase gene sequences of selected perennial ryegrass-associated endophytes and reference isolates. Diagram properties are as described for Figure 1.

**Figure 5:**
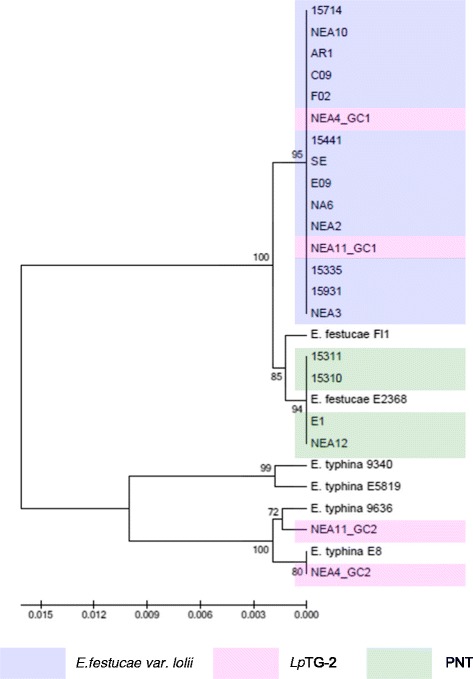
Phylogram resulting from ML analysis of MEAB gene sequences of selected perennial ryegrass-associated endophytes and reference isolates. Diagram properties are as described for Figure 1.

**Figure 6:**
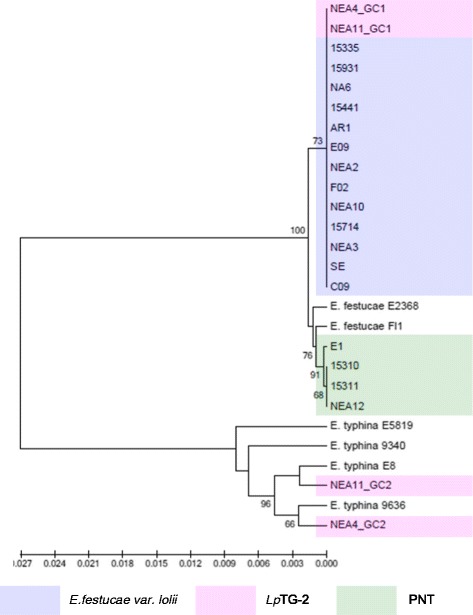
Phylogram resulting from ML analysis of DEAD gene sequences of selected perennial ryegrass-associated endophytes and reference isolates. Diagram properties are as described for Figure 1.

The significant levels of divergence between clades suggest different evolutionary origins. In order to identify potential progenitor genomes among contemporary teleomorphic taxa, phylogenetic analysis was repeated to include representative isolates of *Epichloë* species, and phylograms were constructed using both ML and NJ methods. All trees resulting from phylogenetic analysis using each method were congruent in topology, which indicated similar evolutionary relationships in respect of both *E. festucae* var. *lolii* and *Lp*TG-2 (Figures 1, 2, 3, 4, 5 and 6). Again, separation of PNT strains from the other asexual taxa was supported by >76% bootstrap values in the ML analysis and >73% bootstrap values in the NJ analysis, apart from for *tubB*, which displayed moderate bootstrap support (62% for ML analysis and 66% for NJ analysis).

In general, the single gene copies characteristic of *E. festucae* var. *lolii* and PNT-derived strains were most closely related to those of strains of *E. festucae*. However, contrasting patterns of affinity with specific *E. festucae* isolates were observed between the different selected genes. In the *tubB*-specific phylogram, *E. festucae* strain E2368- and *E. festucae* Fl1-derived sequences exhibited a similar degree of affinity to those of each of the haploid taxa. In contrast, in the *perA*-specific phylogram, *E. festucae* var. *lolii*-derived sequences were grouped more closely with *E. festucae* Fl1, while PNT-derived sequences were located closer to *E. festucae* strain E2368. Furthermore, strain E1 formed a sister group with *E. festucae* strain E2368. In the phylograms derived from the glycosyl hydrolase, MEAB protein and DEAD-box helicase genes, both *E. festucae* Fl1 and *E. festucae* strain E2368 were closer to the PNT genotypes. Finally, a higher similarity between *E. festucae* var. *lolii* and both *E. festucae* strains (E2368 and Fl1) was observed in the *tefA*-based phylogenetic tree.

The genomes of the heteroploid *Lp*TG-2 endophyte contained two distinct copies for each gene, reflecting the hybrid origin of this taxon. While GC1 was closely related to those of *E. festucae* var. *lolii*, GC2 showed affinity to the corresponding sequences from multiple isolates of *E. typhina* related sequences. In all individual gene-based phylograms (with the exception of that derived from the glycosyl hydrolase), as well as the concatenated gene-based tree, *Lp*TG-2 GC2 was more closely related to *E. typhina* E8 (strain ATCC_200736) and *E. typhina* 9636 than the two other *E. typhina* strains, 9340 and E5819. Furthermore, in the *tubB*- and MEAB protein-specific phylograms, GC2 of *Lp*TG-2 strain NEA4 was closer to *E. typhina* E8, while that of NEA11 was closer to *E. typhina* 9636. In contrast, in the DEAD box-based phylogram, NEA11 GC2 was closer to *E. typhina* E8, while NEA4 GC2 was closer to *E. typhina* 9636.

### Identification and phylogenetic characterisation of MT genes

Comparison between the genomic sequences of perennial ryegrass-associated endophytes and reference gene sequences corresponding to the *MT* idiomorphs, *MTA* (*mtaA*, *mtaB*, *mtaC*) and *MTB* (*mtbA*), revealed the presence of a single idiomorph in all strains. *MTB* was detected for all *E. festucae* var. *lolii* and PNT strains, with the single exception of E1, which possessed all three genes corresponding to mating type idiomorph *MTA*. Two copies of *mtbA* were identified for each of the two *Lp*TG-2 strains (Table 2).

**Table 2:** ***MT*** **genes present in perennial ryegrass-associated endophytes**

**Endophyte taxon**	**Strain**	***MT***			
		***MTA***			***MTB***
		***mtaA***	***mtaB***	***mtaC***	***mtbA***
***E. festucae*** **var.** ***lolii***	SE	-	-	-	1
	15335	-	-	-	1
	15441	-	-	-	1
	15714	-	-	-	1
	NEA3	-	-	-	1
	F02	-	-	-	1
	AR1	-	-	-	1
	C09	-	-	-	1
	E09	-	-	-	1
	NA6	-	-	-	1
	NEA10	-	-	-	1
	15931	-	-	-	1
	NEA2	-	-	-	1
**PNT**	15310	-	-	-	1
	15311	-	-	-	1
	E1	1	1	1	-
	NEA12	-	-	-	1
***Lp*** **TG-2**	NEA4	-	-	-	2
	NEA11	-	-	-	2

Phylogenetic analysis was performed by comparison of the *mtbA* gene copies, excluding only strain E1 due to possession of the opposing *MT* idiomorph. In contrast to the other 6 gene-specific phylograms, the *MT* gene-specific tree was separated into two major clades, one representing *E. festucae* var. *lolii*, *Lp*TG-2 GC1 and PNT, the other representing *Lp*TG-2 GC2 (Additional file 2). The *MT* sequences of the first clade were similar, and clustered with that of *E. festucae* Fl1, while those of the second clade were also similar, and showed affinity, albeit at a lower level, to those of several strains of *E. typhina*.

### Phylogenetic characterisation based on concatenated gene sequences

**Figure 7:**
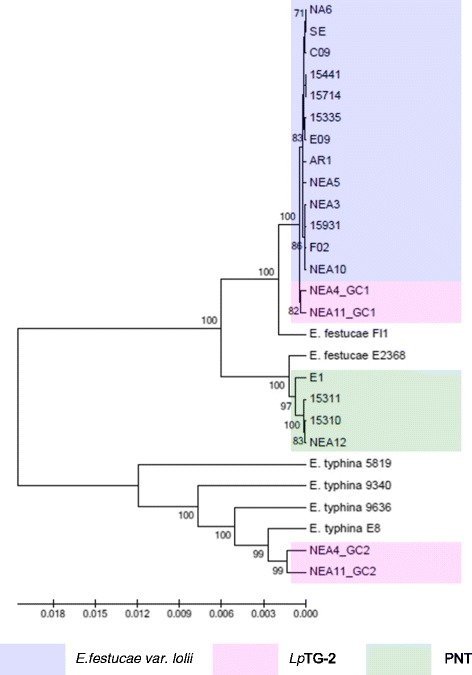
Phylogram resulting from ML analysis of concatenated gene sequences of selected perennial ryegrass-associated endophytes and reference isolates. Diagram properties are as described for Figure 1.

All gene sequences, apart from those of the *MT* genes (due to presence of opposite *MT* idiomorph in E1) were concatenated and analysed by ML and NJ methods, providing strong bootstrap support for all major clades that were defined by single gene-based phylogenetic analysis (Figure 7). In this phylogram, *E. festucae* Fl1 clustered with *E. festucae* var. *lolii,* and *E. festucae* 2368 clustered with PNT. However, when the *perA* sequences were removed from the concatenated gene structure, both *E. festucae* Fl1 and *E. festucae* E2368 clustered preferentially with PNT (Additional file 3), suggesting a predominant influence of *perA* in the analysis, possibly due to the large length of this gene in comparison to the others (Table 3).

**Table 3:** **Lengths of genes used for construction of phylogenetic tree based on concatenated gene sequences**

**Gene**	**Length of gene (bp)**
***tefA***	1882
***tubB***	2359
***perA***	8505
**DEAD/DEAH helicase**	1990
**Glycosyl hydrolase**	2490
**MEAB**	1623

## Discussion

### Phylogenetics of previously characterised endophyte taxa

In the present study, sequence diversity of multiple independently selected genes was used to identify the phylogenetic affinities between perennial ryegrass-associated endophytes, and to interpret potential progenitor relationships with contemporary sexual *Epichloë* species. This study represents the first use of whole genome data to obtain full-length sequence of multiple genes for phylogenetic analysis of perennial ryegrass-associated endophytes. The results revealed the existence of three major groups, confirming relationships previously deduced from SSR-based genotyping, of which two (corresponding to *E. festucae* var. *lolii* and *Lp*TG-2) have been previously described. Limited sequence diversity for all selected genes was detected within each taxon, as would be expected for asexual species [46]. In contrast, the multiple isolates of the sexual species *E. typhina* displayed higher levels of variation, as is further supported by possession of different *MT* genes and substantial differences between mitochondrial genomes [24,30].

The identities of progenitors for *E. festucae* var. *lolii* and *Lp*TG-2 have been identified in several previous phylogenetic studies. The most common endophyte of perennial ryegrass, *E. festucae* var. *lolii,* has been proposed as a direct haploid descendant of *E. festucae*, while the heteroploid *Lp*TG-2 was suggested to have evolved through hybridisation of *E. festucae* var. *lolii* and *E. typhina* [24,47]. Equivalent phylogenetic affinities were observed in the current study. Of the two reference *E. festucae* strains that were used, Fl1 appears to be more closely related to *E. festucae* var. *lolii* strains based on the *tubB-* and *perA*-specific phylograms, while both strains exhibited similar levels of affinity to *E. festucae* var. *lolii* in the *tefA*-derived tree.

The origin of heteroploid endophytes may be verified by comparison with multiple candidate progenitor species [48]. *E. typhina* E8, which was isolated from perennial ryegrass, provided the closest match to *Lp*TG-2 GC2 in the phylograms based on both individual and concatenated gene sequence, consistent with a previous study [24]. The higher variability of GC2 as compared to GC1 may provide evidence for the contributions of different *E. typhina* lineages to the origin of *Lp*TG-2 strains, rather than a monophyletic event. Further evidence for complexity in the evolution of *Lp*TG-2 is apparent from the *MT* gene-based analysis. Of the *E. typhina* strains that were subjected to analysis, *E. typhina* 9340 and *E. typhina* MAFF (AB161000.1) display the *MTB* idiomorph, while the opposing (*MTA*) mating type was observed in *E. typhina* E8. Hence, despite the close relationships between *E. typhina* E8 and *Lp*TG-2 GC2 revealed by analysis based on other nuclear genes, a lineage similar to, but distinct from *E. typhina* E8 must have been the direct ancestor of the *Lp*TG-2 strains included in the present study.

### Phylogenetic identities of PNT genotypes

Similar patterns of relationships between the three distinct clades were apparent for all independently selected gene sequences, apart from the *MT* genes. The PNT individuals were separated from the other two taxa with strong bootstrap support in all phylograms that included sexual *Epichloë* reference isolates, except for the moderate value obtained for the *tubB*-based tree. A similar difference between confidence of structure obtained with individual genes was also observed during phylogenetic analysis of annual ryegrass-derived endophytes based on *tubB* and nuclear ribosomal DNA internal transcribed spacer (rDNA-ITS) sequences. In this analysis, many branches of the rDNA-ITS-based phylogram displayed a lower bootstrap support than the *tubB*-based phylogram. The discrepancy was interpreted in terms of homoplasy effects and/or the lower number of informative characters that are available from rDNA-ITS gene sequence [26]. Similar considerations may apply to the variability of bootstrap support observed in the present study.

Convergence of gene structure between otherwise distantly related genotypes may also apply to the highly conserved *tefA* gene, and account for the anomalous location of strain E1 in the respective phylogram. Equivalent patterns of relationships have been reported in other phylogenetic analysis studies. For instance, the *tefA* gene of the meadow fescue-derived endophyte *N. uncinatum* was most closely related to that of *E. bromicola*, but the single *tubB* gene showed a closer relationship to that of *E. typhina* [48]. To address such discrepancies, the concatenation approach has been developed in order to obtain more accurate phylogenetic results [49]. Interestingly, the concatenated sequence-based phylograms in the present study displayed strong bootstrap support for the presence of a third taxon grouping, providing additional confidence in the inferences from individual gene sequence analysis.

As for *E. festucae* var. *lolii*, PNT strains exhibit the closest relationships to *E. festucae* among the available contemporary sexual *Epichloë* species. However, in respect to strain diversity within *E. festucae*, different relationships were observed across different genes. On the basis of the primary concatenated phylogram, the PNT group showed a closer relationship to *E. festucae* 2368, while *E. festucae* var. *lolii* was grouped with *E. festucae* Fl1. However, this seems likely to be due to the influence of the *perA* gene, which contributes a length of c. 7.3 kb that is common to all strains in the study, close to the combined length of the other 5 genes (c. 10.3 kb). In the absence of the *perA* gene, members of the PNT group seem to exhibit a slightly higher affinity to both *E. festucae* isolates (Additional file 3), perhaps indicating an origin from a similar progenitor, while *E. festucae* var. *lolii* may have arisen from a variant of *E. festucae* that either has not been included in the analysis, or is not represented in the contemporary range of intraspecific diversity. Nonetheless, the *MT* gene-based analysis indicates that all *E. festucae* var. *lolii* and *Lp*TG-2 strains display the *MTB* idiomorph, either in single or double copy number, similar to *E. festucae* Fl1. It is worthwhile to consider whether the conflicting results may be an artefact of limited sampling of genomic diversity, and a definitive analysis will depend on comparison of whole genome sequences.

Separate taxonomic status for members of the PNT group was also supported by evidence from chemotypic diversity. Metabolic profiling for the presence of known alkaloids revealed that all of the *E. festucae* var. *lolii* and *Lp*TG-2 strains included in the present study produce at least one of the three common compounds, lolitrem B, ergovaline and peramine [50]. However, members of the PNT group have been shown to produce none of these metabolites, but rather to synthesise another class of bioactive metabolites, the epoxy-janthitrems [35,51]. The failure to synthesise peramine is consistent with the 1223 bp 3’-terminal deletion of the *perA* gene in all individuals, as well as the premature stop codon at coordinate 2923 bp in all but E1, in each case generating a non-functional gene copy. The presence of the majority of the *perA* gene, however, suggests that PNT endophytes may have possessed an intact *perA* gene at some stage of evolution, followed by a deletion event similar to that inferred to have occurred in endophyte strains *E. typhina* E1022 and *E. festucae* E2368 [28].

## Conclusion

Although the endophyte strains located within the PNT group are phylogenetically distinct from both *E. festucae* var. *lolii* and *Lp*TG-2, evidence suggests that they may represent not one but two distinct novel taxa. Strains 15310, 15311 and NEA12 show near-identity in all individual gene-based trees, indicating a low level of intra-group diversity, and reflecting a probable recent origin from an *E. festucae*-like progenitor with a *MTB* idiomorph. Deletion of the 3’-terminus of *perA* is diagnostic of this group. For these reasons, it is proposed that such members of the PNT group are now designated as belonging to a new taxon, *Lp*TG-3. In contrast, E1, although a sole representative and obviously closely related to *Lp*TG-3, shows unique features, not least the premature stop codon that is additional to the deletion in the *perA* gene, and most significantly, a *MTA* idiomorph. On the latter basis, a common origin with *Lp*TG-3 followed by divergence of asexual lineages is difficult to defend. In contrast, a more parsimonious explanation may involve the origin of both types from different genotypes (with contrasting *MT* idiomorphs) of an *E. festucae*-like progenitor which had already undergone the *perA* deletion, leading to establishment of two parallel lineages of asexual endophyte, further modification of *perA* being confined to the *Lp*TG-3 genomes. Consequently, it is further proposed that although E1 did not originate from a perennial ryegrass host, it is provisionally assigned (pending further investigation) to a fourth taxonomic grouping of perennial ryegrass-associated endophytes as a sister group to *Lp*TG-3, to be designated *Lp*TG-4. As both of the proposed new taxa are capable of producing the novel alkaloid class of epoxy-janthitrems, which display valuable feeding deterrence effects toward invertebrate herbivores, the definition of genomic properties of such taxa is capable of assisting future targeted endophyte discovery programs.

## Availability of supporting data

The data sets supporting the results of this article are included within the article and its additional files. All gene sequences have been deposited in GenBank and sequence alignments are availabe in the TreeBASE repository (http://purl.org/phylo/treebase/phylows/study/TB2:S17154) [52]. Details of GenBank accession numbers are provided for each gene-by-isolate combination in tabular form in Additional file 4.

## Additional files

Additional file 1:
***De novo*** **assembly statistics for sequenced perennial ryegrass-associated endophyte genomes.**
Additional file 2:
**Phylogram resulting from ML analysis of the** ***MT*** **gene sequence,** ***mtbA*** **, of selected perennial ryegrass-associated endophytes and reference isolates.** The GenBank accession numbers of the *MT* genes derived from ryegrass-associated endophytes are provided in Additional file 4. Diagram properties are as described for Figure 1. Additional file 3:
**Phylogram resulting from ML analysis of concatenated gene sequence (** ***tubB*** **,** ***tefA*** **, DEAD, glycosyl hydrolase and MEAB) of selected perennial ryegrass-associated endophytes.** Diagram properties are as described for Figure 1. Additional file 4:
**Genbank accession numbers of the sequences represented in the phylogenetic study.**

## Abbreviations

BLAST: Basic local alignment search tool
*Lp*TG: *Lolium perenne* taxonomic group
LT: Low-throughput
ML: Maximum likelihood
*MT*: Mating type
NCBI: National Centre for Biotechnology Information
NJ: Neighbour joining
PNT: Putative novel taxon
SSR: Simple sequence repeat
