# Supplementary material for: Phylogenomics of asexual Epichloë fungal endophytes forming associations with perennial ryegrass
Source: BMC Evol Biol. 2015 Apr 24;15:72. doi: 10.1186/s12862-015-0349-6 (PMC4458015; doi:10.1186/s12862-015-0349-6)
Supplement: Additional file 1: — De novo assembly statistics for sequenced perennial ryegrass-associated endophyte genomes. [file 12862_2015_349_MOESM1_ESM.docx]

Additional File 1

| **Species/Taxon** | **Strain/Isolate ID** | **Total number**  **of contigs** | **n50** **(bp)** | **Total number of bases**  **in contigs (bp)** | **Length of longest**  **contig (bp)** |
| --- | --- | --- | --- | --- | --- |
| *E. festucae* var. *lolii* | SE | 17519 | 13760 | 31286952 | 140010 |
|  | 15335 | 40273 | 5834 | 30096989 | 56820 |
|  | 15441 | 38878 | 17712 | 30164941 | 126092 |
|  | 15714 | 43298 | 5290 | 29780677 | 67599 |
|  | NEA3 | 32491 | 15713 | 28926318 | 119559 |
|  | F02 | 30274 | 19575 | 30619879 | 168025 |
|  | AR1 | 35418 | 26352 | 30343418 | 208982 |
|  | C09 | 40726 | 26828 | 30318654 | 193771 |
|  | E09 | 36729 | 9053 | 28901364 | 71582 |
|  | NA6 | 38117 | 31066 | 29988031 | 194340 |
|  | NEA10 | 31664 | 25016 | 29423139 | 142283 |
|  | 15931 | 31588 | 31918 | 28593996 | 151816 |
|  | NEA2 | 39342 | 12274 | 38225667 | 132070 |
| PNT | 15310 | 37525 | 32925 | 29918953 | 205383 |
|  | 15311 | 36027 | 38107 | 29907095 | 184116 |
|  | NEA12 | 39966 | 6160 | 29995801 | 130138 |
|  | E1 | 38274 | 15078 | 30630415 | 136326 |
| *Lp*TG-2 | NEA4 | 114660 | 1407 | 65330778 | 20182 |
|  | NEA11 | 64212 | 456 | 24886670 | 12442 |
