# Supplementary figures and images for: Phylogenomics of asexual Epichloë fungal endophytes forming associations with perennial ryegrass
Source: BMC Evol Biol. 2015 Apr 24;15:72. doi: 10.1186/s12862-015-0349-6 (PMC4458015; doi:10.1186/s12862-015-0349-6)

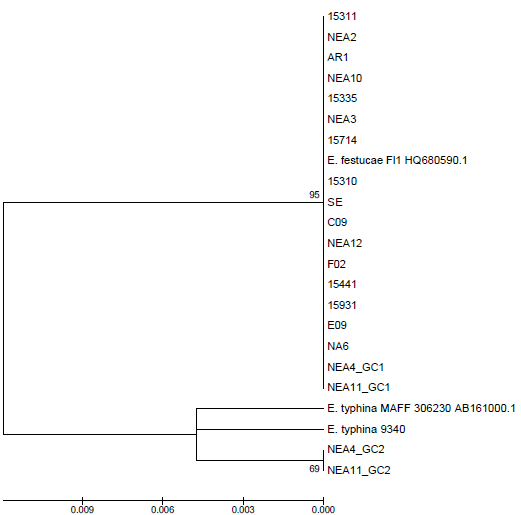


*E.festucae var. lolii*

*Lp*TG-2

PNT

**Additional File 2**

Supplement: Additional file 2: — Phylogram resulting from ML analysis of the MT gene sequence, mtbA , of selected perennial ryegrass-associated endophytes and reference isolates. The GenBank accession numbers of the MT genes derived from ryegrass-associated endophytes are provided in Additional file 4. Diagram properties are as described for Figure 1. [file 12862_2015_349_MOESM2_ESM.docx]

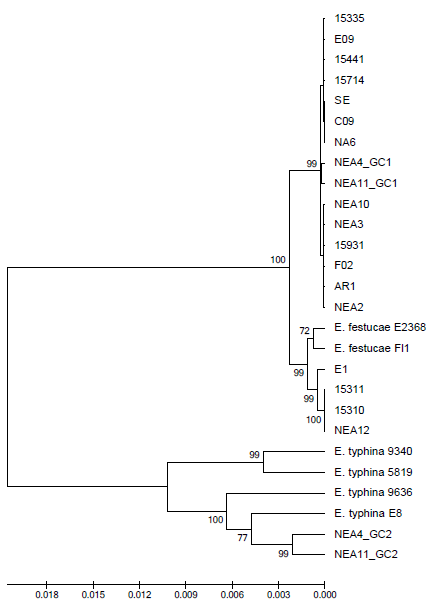


*E.festucae var. lolii*

*Lp*TG-2

PNT

**Additional File 3**

Supplement: Additional file 3: — Phylogram resulting from ML analysis of concatenated gene sequence ( tubB , tefA , DEAD, glycosyl hydrolase and MEAB) of selected perennial ryegrass-associated endophytes. Diagram properties are as described for Figure 1. [file 12862_2015_349_MOESM3_ESM.docx]
