# Supplementary material for: Phylogenomics of asexual Epichloë fungal endophytes forming associations with perennial ryegrass
Source: BMC Evol Biol. 2015 Apr 24;15:72. doi: 10.1186/s12862-015-0349-6 (PMC4458015; doi:10.1186/s12862-015-0349-6)
Supplement: Additional file 4: — Genbank accession numbers of the sequences represented in the phylogenetic study. [file 12862_2015_349_MOESM4_ESM.docx]

**Additional File 4** GenBank accession numbers of the sequences represented in the phylogenetic study.

| **Species/Taxon** | **Strain/**  **isolate ID** | **Accession numbers** | | | | | | | | | |
| --- | --- | --- | --- | --- | --- | --- | --- | --- | --- | --- | --- |
|  |  | ***tefA*** | ***tubB*** | ***perA*** | **DEAD/DEAH box helicase (Sbp4)** | **Glycosyl hydrolase** | **MEAB** | **MT** | | | |
|  |  |  |  |  |  |  |  | *mtBA* | *mtAA* | *mtAB* | *mtAC* |
| ***E. festucae* var. *lolii*** | SE | KP834565 | KP834586 | KP834538 | KP834456 | KP834479 | KP834519 | KP834499 | - | - | - |
|  | 15335 | KP834548 | KP834584 | KP834527 | KP834441 | KP834462 | KP834506 | KP834483 | - | - | - |
|  | 15441 | KP834549 | KP834569 | KP834528 | KP834442 | KP834463 | KP834507 | KP834484 | - | - | - |
|  | 15714 | KP834550 | KP834570 | KP834529 | KP834443 | KP834464 | KP834508 | KP834485 | - | - | - |
|  | NEA3 | KP834557 | KP834576 | KP834536 | KP834450 | KP834471 | KP834515 | KP834491 | - | - | - |
|  | F02 | KP834545 | KP834566 | KP834524 | KP834438 | KP834459 | KP834503 | KP834480 | - | - | - |
|  | AR1 | KP834552 | KP834572 | KP834531 | KP834445 | KP834466 | KP834510 | KP834487 | - | - | - |
|  | C09 | KP834553 | KP834573 | KP834534 | KP834446 | KP834467 | KP834511 | KP834488 | - | - | - |
|  | E09 | KP834554 | KP834574 | KP834532 | KP834448 | KP834469 | KP834513 | KP834489 | - | - | - |
|  | NA6 | KP834556 | KP834575 | KP834533 | KP834449 | KP834470 | KP834514 | KP834490 | - | - | - |
|  | NEA10 | KP834561 | KP834580 | KP834535 | KP834453 | KP834475 | KP834517 | KP834495 | - | - | - |
|  | 15931 | KP834551 | KP834571 | KP834530 | KP834444 | KP834465 | KP834509 | KP834486 | - | - | - |
|  | NEA2 | KP834560 | KP834579 | KP834537 | KP834452 | KP834474 | KP834516 | KP834494 | - | - | - |
| ***Lp*TG*-*2** | NEA4_GC1 | KP834559 | KP834577 | KP834541 | KP834457 | KP834472 | KP834520 | KP834492 | - | - | - |
|  | NEA4_GC2 | KP834558 | KP834578 | KP834542 | KP834451 | KP834473 | KP834521 | KP834493 | - | - | - |
|  | NEA11_GC1 | KP834562 | KP834581 | KP834543 | KP834458 | KP834476 | KP834522 | KP834496 | - | - | - |
|  | NEA11_GC2 | KP834563 | KP834582 | KP834544 | KP834454 | KP834477 | KP834523 | KP834498 | - | - | - |
| ***Lp*TG*-*3** | 15310 | KP834546 | KP834567 | KP834525 | KP834439 | KP834460 | KP834504 | KP834481 | - | - | - |
|  | 15311 | KP834547 | KP834568 | KP834526 | KP834440 | KP834461 | KP834505 | KP834482 | - | - | - |
|  | NEA12 | KP834564 | KP834583 | KP834540 | KP834455 | KP834478 | KP834518 | KP834497 | - | - | - |
| ***Lp*TG*-*4** | E1 | KP834555 | KP834585 | KP834539 | KP834447 | KP834468 | KP834512 | - | KP834500 | KP834501 | KP834502 |
